# Nationwide web survey on implementing the 2023 ETA guidelines for second-line management thyroid nodules with atypia of undetermined significance

**DOI:** 10.1007/s42000-025-00698-4

**Published:** 2025-07-28

**Authors:** Eleni Sazakli, Olga Karapanou, Gerasimos P. Sykiotis, Katerina Saltiki, Marina Michalaki

**Affiliations:** 1 Faculty of Medicine, School of Health Science, University Hospital of Patras, Patras, PC26500 Greece; 2 Endocrine Department, 401, General Military Hospital of Athens, Athens, 11527 Greece; 3 Service of Endocrinology, Diabetology and Metabolism, Lausanne University Hospital and University of Lausanne, Lausanne, 1011 Switzerland; 4 Endocrine Unit, Department of Clinical Therapeutics, National and Kapodistrian University, Athens, 11528 Greece

**Keywords:** Survey, Thyroid nodules, 2023 ETA guidelines, EU-TIRADS 4–5, AUS, Repeat FNA

## Abstract

**Background:**

Thyroid nodules affect up to 70% of adults undergoing ultrasonography. The European Thyroid Association (ETA) recently issued high-quality guidelines outlining management options. However, their implementation in routine practice remains uncertain. This study examined guideline adoption and barriers, focusing on nodules with atypia of undetermined significance (AUS), a particularly challenging cytological category.

**Methods:**

From November 2023 to April 2024, members of the Hellenic Endocrine Society (HES) participated in a web-based survey concerning management of a 2.5 cm thyroid nodule with fine-needle aspiration (FNA) cytology results of AUS. The scenario varied based on the nodule’s sonographic risk, namely, as intermediate (EU-TIRADS 4) or high (EU-TIRADS 5). The questionnaire also collected demographic information and inquired about reasons for non-adherence to the guidelines.

**Results:**

The response rate was 25%. For an EU-TIRADS 4 nodule, 61% chose to repeat FNA, whereas only 23% would repeat FNA for an EU-TIRADS 5 nodule, despite the 2023 ETA guidelines recommending this approach as the next step in both cases. More experienced endocrinologists were less likely to opt for repeat FNA and more likely to choose total thyroidectomy in the first scenario, whereas experience did not influence preferences in the second scenario. Reasons for non-compliance were skepticism regarding the recommendations, limited access to reliable neck ultrasonography, and molecular testing, and a shortage of high-volume surgeons.

**Conclusions:**

Greek endocrinologists deviate from the 2023 ETA guidelines for the management of thyroid nodules with AUS cytology in daily clinical practice. These findings highlight the need for targeted educational strategies and enhancement of clinical infrastructure in Greece.

**Supplementary Information:**

The online version contains supplementary material available at 10.1007/s42000-025-00698-4.

## Introduction

The incidence of thyroid nodules has reached epidemic rates in recent years owing to the widespread use of thyroid ultrasonography, leading to the detection of asymptomatic thyroid nodules in up to 70% of cases [1–4]. Therefore, a substantial number of patients require further evaluation. Scientific societies have published guidelines for the management of thyroid nodules to avoid unnecessary diagnostic procedures and minimize overtreatment, as approximately 90–95% of thyroid nodules are benign [5–7].

Recently, the European Thyroid Association (ETA) released high-quality clinical guidelines for the management of thyroid nodules [6]. According to these guidelines, the threshold for fine-needle aspiration (FNA) is based on the nodule’s diameter and its imaging characteristics, according to the European Thyroid Imaging Reporting and Data System (EU-TIRADS) system [6]. The cytology category of FNA specimens, classified by the Bethesda System for Reporting Thyroid Cytopathology (TBSRTC), guides the therapeutic decision-making process.

However, the extent to which the 2023 ETA clinical guidelines are followed in daily clinical practice is unknown. Our daily interactions reveal that many colleagues continue to follow outdated practices rather than adopting new, evidence-based approaches.

To address this issue—and to explore the underlying reasons for potential non-adherence —we conducted a web-based survey among endocrinologists in Greece (members of the Hellenic Endocrine Society, HES). Given that the management of thyroid nodules with diagnostic category of atypia of undetermined significance (AUS), according to the 3rd edition of the BETHESDA system, is particularly challenging [8–11], we focused our study on thyroid nodules with AUS cytology. The specific aims were as follows: (i) to evaluate adherence to the 2023 ETA clinical practice guidelines for the management of thyroid nodules classified as Bethesda AUS [6]; and (ii) to explore reasons for non-adherence, if any.

## Materials and methods

### Study design and participant recruitment

The study participants were endocrinologists and HES members. According to HES records, there are currently 809 endocrinologists in Greece. The target sample size was 20% of the total population (*n* = 162), as calculated by the adjusted Cochran’s formula for finite populations. This target was based on an estimated population of 810 endocrinologists, using a confidence level of 85% and a 5% margin of error. Ethics approval was granted by the Ethics Committee of the University of Patras (approval 15770/28-07-2023). Participation was voluntary. The study was conducted in accordance with the guidelines of the Declaration of Helsinki, as revised in 2013.

We employed a web-based survey constructed using SurveyLegend, a survey web application that ensured anonymous collection of responses and automatic exclusion of repeat submissions from the same IP address. An announcement of the survey briefly describing the study and its objectives, including the link to the electronic platform with the questionnaire, was sent by email to HES, who in turn notified all their members. Potential responders were assured that participation was voluntary and that confidentiality and anonymity would be respected. Contact information from the corresponding author was also provided for possible clarifications. Recruitment took place from November 6, 2023, to April 15, 2024. An electronic reminder was sent to HES members on January 24, 2024. A pilot survey was conducted with a small group (*n* = 15) of endocrinologists before the survey was launched. The questionnaire was repeated 5 months later to assess its repeatability through Cohen’s kappa test of agreement. Responses collected in this pilot phase of test-retest were not included in data processing.

### Questionnaire design

The survey items were selected by the researchers and the questionnaire was administered in Greek, as it was distributed to members of the HES. An English translation of the questionnaire is available in the Supplemental Data.

The questionnaire consisted of three parts. The first part included seven questions about the respondents’ demographic data (sex, age, occupational status, geographical region, and years of experience), self-reported confidence in the diagnosis and treatment of patients with thyroid nodules or cancer (on a 5-point Likert scale), and their choice of the most helpful educational tools for managing thyroid nodules (conferences, clinical practice, or literature).

The second part of the survey presented 12 clinical scenarios, grouped into the following clusters based on the clinical issue addressed: (i) second-line management of thyroid nodules with AUS; (ii) management of low-risk papillary thyroid microcarcinoma (this cluster has already been published) [12] and (iii) management of low- to intermediate-risk papillary thyroid carcinoma. Each scenario required endocrinologists to choose one of four management approaches. Herein, we present the first cluster of clinical scenarios, which includes two nearly identical cases addressing second-line management of thyroid nodules with AUS cytology, as shown in Box 1 (Supplementary Data).

**Table:** 

Box 1
1. A 65-year-old woman has a non-functioning solitary thyroid nodule with a maximum diameter of 2.5 cm and an ultrasound pattern classified as EU-TIRADS 4 (risk of malignancy: 6–17%). Cytology revealed atypia of undetermined significance (Bethesda category III) according to The Bethesda System for Reporting Thyroid Cytopathology), 2nd edition. What is your next step? (A) repetition of FNA; (B) molecular testing (if available); (C) lobectomy; (D) total thyroidectomy
2. A 65-year-old woman has a non-functioning solitary thyroid nodule, with a maximum diameter of 2.5 cm and an ultrasound pattern classified as EU-TIRADS 5 (risk of malignancy: 26–87%). Cytology revealed atypia of undetermined significance (Bethesda category III) according to The Bethesda System for Reporting Thyroid Cytopathology), 2nd edition. What is your next step? (A) repetition of FNA; (B) molecular testing (if available); (C) lobectomy; (D) total thyroidectomy

The third part of the questionnaire included a final question addressing the main reasons for non-adherence to guidelines across all 12 clinical scenarios, without being limited to the 2023 ETA guidelines for thyroid nodule management. All participants were asked and five possible answers were offered with the option of selecting one or more. The choices were as follows: (i) insufficient information, (ii) skepticism about the guidelines and concerns for patient safety, or (iii) inability to perform a reliable neck ultrasound, d. inability to conduct molecular testing, and e. lack of experienced surgeons across Greece.

### Statistical data analysis

Descriptive statistics were computed to summarize the respondents’ demographic data. The group proportions were calculated for categorical variables. Differences in proportions were tested using the χ^2^ test. The non-parametric Kolmogorov–Smirnov test was employed to compare the geographical distributions of the entire population and the participants’ sample. The reliability of the questionnaire was assessed using Cohen’s Kappa coefficient, a measure of agreement based on the responses of the group of endocrinologists during the pilot phase at two time points. Multivariate binary (each response choice versus all other available choices) logistic regression models were employed to assess the association between the selected answer and the participants’ characteristics: sex, years since residency (ordinal variable), clinician practice location (large cities vs. rural areas), employment sector, and self-confidence level. Logistic models were constructed with the forward stepwise (likelihood ratio) method and factors with *p* < 0.150 were included in the final models. The statistical significance level was set at *p* = 0.05. Statistical analysis was performed using IBM SPSS version 28 (IBM Corp., Armonk, NY, USA).

## Results

### First part: study participants and their characteristics

A total of 201 of 809 HES members participated in the study (response rate of 25%), thus exceeding the target sample size of 162. The demographic characteristics of the participants are presented in Table 1. The sex distribution observed in our respondent sample closely mirrored that of the total population of Greek endocrinologists as recorded in the HES registry, supporting the representativeness of our sample. Additionally, their geographical distribution matched the real distribution of Greek endocrinologists (Kolmogorov-Smirnov Z test, *p* = 1.000). The interrater reliability of the pilot phase of the questionnaire was satisfactory (kappa = 0.804, *p* < 0.001).

**Table 1:** Demographic characteristics of the study population (endocrinologists)

		Percentage (%) *N* = 201
Sex	Male	42.0
	Female	58.0
Age (range, in years)	30–39	10.4
	40–49	31.3
	50–59	35.8
	60–69	18.9
	> 70	3.5
Employed in	Public sector	23.4
	Private sector	76.6
Years since residency	1–5	17.9
	6–10	16.4
	11–30	56.7
	> 30	9.0
Geographical distribution	Large cities (Athens, Thessaloniki)	68.2
	Other areas	31.8
Level of self-confidence in managing patients with thyroid nodules or cancer	Moderately confident	27.0
	Very confident	61.5
	Absolutely confident	11.5

The majority of Greek endocrinologists (61.5%) reported being self-confident in managing patients with thyroid nodules or papillary thyroid carcinoma (Table 1). When asked about the most helpful educational tool for managing these conditions, 33.8% selected all three available options, namely, clinical practice and literature—while 26.3% chose clinical practice alone.

### Second part: responses to the clinical scenarios

**Fig. 1:**
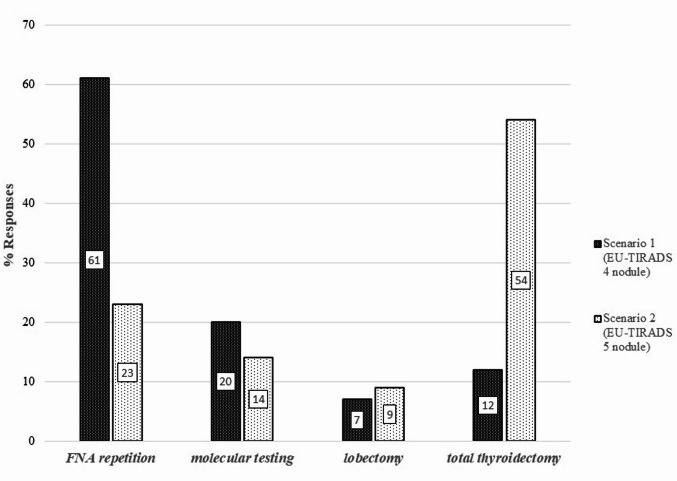
Distribution of physicians’ responses to two clinical scenarios about thyroid nodules. Notably, the 2023 ETA clinical practice guidelines for managing thyroid nodules [6] recommend FNA repetition for both scenarios.

*First scenario*:

A total of 122 physicians (61.0%) chose repeat FNA as the best treatment for an EU-TIRADS 4 nodule with Bethesda III-AUS cytology (Fig. 1). Clinician practice location (large cities vs. other areas) was not associated with the responses (Table 2). Physicians with more years of experience (> 10 years) were less likely to choose repeat FNA than those with < 5 years of experience. Concerning the choice of molecular testing, which was selected by 20% of the participants, the higher the self-confidence level, the higher the odds of giving this answer. Lobectomy was chosen by only 7% of participants, with women being 10 times more likely than men to choose this option. Experience appeared to influence this choice: compared to novice physicians with < 5 years since residency, physicians 11–30 years post-residency were 5.91 times more likely to select total thyroidectomy, and those with > 30 years since residency were even more likely (12.69 times) to make this choice.

**Table 2:** Associations of selection of responses with study population characteristics

Scenario 1					
***A) FNA repetition***	repeat FNA	other response	**O.R.** ^**1**^	**95% C.I.** ^**2**^	***p-value***
Years since residency	*n*	*n*			
1–5	*26*	*8*	1		
6–10	*24*	*9*	0.82	0.27–2.47	*0.725*
11–30	*63*	*49*	**0.40**	**0.17–0.95**	***0.038***
> 30	*7*	*11*	**0.20**	**0.06–0.67**	***0.010***
Sex					*0.215*
Employment sector					*0.256*
Geographical area of employment					*0.456*
Self-confidence level					*0.958*
***B) Molecular testing***	Molecular test	other response	**O.R.**	**95% C.I.**	***p-value***
Self-confidence level	*n*	*n*			
Moderately confident	*5*	*48*	1		
Very confident	*29*	*92*	**3.03**	**1.10–8.32**	***0.032***
Absolutely confident	*6*	*17*	**3.39**	**0.92–12.55**	***0.068***
Sex					*0.987*
Years since residency					*0.937*
Employment sector					*0.698*
Geographical area of employment					*0.808*
***C) Lobectomy***	Lobectomy	other response	**O.R.**	**95% C.I.**	***p-value***
Sex	*n*	*n*			
Male	*1*	*81*	1		
Female	*12*	*103*	**10.60**	**1.34–83.67**	***0.025***
Employment sector					
Public	*1*	*46*	1		
Private	*12*	*138*	**4.98**	**0.62–39.90**	***0.130***
Years since residency					*0.607*
Geographical area of employment					*0.685*
Self-confidence level					*0.963*
***D) Total thyroidectomy***	Thyroidectomy	other response	**O.R.**	**95% C.I.**	***p-value***
Years since residency	*n*	*n*			
1–5	*1*	*33*	1		
6–10	*1*	*32*	1.03	0.06–17.20	*0.983*
11–30	*17*	*95*	**5.91**	**0.76–46.12**	***0.090***
> 30	*5*	*13*	**12.69**	**1.35–119.33**	***0.026***
Sex					*0.769*
Employment sector					*0.278*
Geographical area of employment					*0.155*
Self-confidence level					*0.928*
Scenario 2					
***A) FNA repetition***	repeat FNA	other response	**O.R.**	**95% C.I.**	***p-value***
Employment sector	*n*	*n*			
Private	*30*	*120*	1		
Public	*16*	*31*	**2.07**	**1.00–4.26**	***0.050***
Sex					*0.385*
Years since residency					*0.636*
Geographical area of employment					*0.169*
Self-confidence level					*0.170*
***B) Molecular testing***					
Sex					*0.347*
Years since residency					*0.463*
Employment sector					*0.830*
Self-confidence level					*0.434*
Geographical area of employment					*0.823*
***C) Lobectomy***	Lobectomy	other response	**O.R.**	**95% C.I.**	***p-value***
Sex	*n*	*n*			
Male	*10*	*72*	1		
Female	*7*	*108*	**0.42**	**0.15–1.16**	***0.094***
Geographical area of employment					
Other areas	*1*	*61*	1		
Large cities	*16*	*119*	**8.94**	**1.15–69.56**	***0.036***
Years since residency					*0.671*
Employment sector					*0.372*
Self-confidence level					*0.593*
***D) Total thyroidectomy***	Thyroidectomy	other response	**O.R.**	**95% C.I.**	***p-value***
Employment sector	*n*	*n*			
Private	*88*	*62*	1		
Public	*19*	*28*	**0.48**	**0.25–0.93**	***0.030***
Sex					*0.319*
Years since residency					*0.587*
Self-confidence level					*0.191*
Geographical area of employment					*0.976*

^1^O.R.: odds Rratio, ^2^C.I.: confidenceinterval. Significant associations are depicted in bold.

*Second scenario*:

For an EU-TIRADS 5 nodule with Bethesda III-AUS cytology, 108 physicians (54.0%) chose total thyroidectomy (Fig. 1). In contrast to the first scenario, neither years since residency nor self-confidence were associated with the answers (Table 2). Endocrinologists working in the public sector were less likely to prefer total thyroidectomy and had 2.07 times higher odds of choosing FNA repetition than their peers in the private sector. Molecular testing was preferred by 14% of participants. Lobectomy was chosen by 7% of participants, with physicians working in large cities (Athens and Thessaloniki) having 8.94 times higher odds of choosing lobectomy over other options than those working in other areas. In contrast to the first scenario, women tend to be less likely than men to select lobectomy, but this association was not significant (Table 2).

### Third part: question “which are the main reasons for non-compliance with the guidelines?”

Ninety-four endocrinologists (46.8%) expressed skepticism about the guidelines provided by scientific societies and highlighted concerns about patient safety. Inability to perform reliable ultrasound and molecular testing were cited by 71 (35.3%) and 69 (34.3%) endocrinologists, respectively, as reasons for non-compliance, followed by lack of proper information (22.4%) and shortage of experienced surgeons (17.9%). Physicians not working in Attica (where the capital city, Athens, is situated) considered the lack of experienced surgeons as a reason for non-adherence to the guidelines more than physicians working in Attica (χ^2^ test, *p* = 0.009).

## Discussion

To the best of our knowledge, this is the first web-based survey exploring endocrinologists’ adherence to the 2023 ETA Clinical Practice Guidelines for thyroid nodule management. We surveyed the approach to two nearly identical clinical scenarios involving a 2.5 cm solitary nonfunctioning thyroid nodule in a middle-aged woman with an FNA cytology report of Bethesda III-AUS, but with differing ultrasound characteristics and associated sonographic risk of malignancy (ROM). Our sample was representative of the national population of endocrinologists in Greece and of sufficient size, owing to a solid response rate of 25%, comparable to other surveys [13, 14].

The main and most intriguing finding is the substantial deviation from the recently released ETA guidelines. The EU-TIRADS category significantly influenced the clinical decisions of the respondents and took precedence over cytology results. For a nodule classified as EU-TIRADS 4, 61% of endocrinologists opted for repeat FNA, whereas for a nodule classified as EU-TIRADS 5, 54% favored total thyroidectomy, with only 23% choosing repeat FNA. This approach deviates from the recommendations of not only the ETA, but also of several other scientific societies [5, 7, 15, 16]. The recent 2023 ETA guidelines [6] mandate repeat FNA in these clinical scenarios regardless of the EU-TIRADS category. The primary reasons cited for not adhering to the guidelines were skepticism about them, as well as the lack of access to reliable neck ultrasonography and molecular testing nationwide. Lack of proper information and of experienced surgeons is frequently invoked.

Few surveys have examined clinical practices in the management of thyroid nodules. One American web-based survey, conducted in 2015 and completed by 897 members of the Endocrine Society, AACE, and ATA, found that for AUS/FLUS cytology, 31.5% of respondents opted for repeat FNA, 38.8% for molecular testing, and 24.4% referred patients for thyroid surgery [17]. It is important to note that this survey was conducted immediately prior to the release of the 2015 ATA guidelines.

Repeat FNA may significantly reduce the number of unnecessary surgeries. In nodules with AUS cytology, repeated FNA yields benign results in 36–50% of cases [10, 18]. In our study, FNA repetition was preferred by most participants (61%) for an EU-TIRADS 4 nodule, but only by 23% for an EU-TIRADS 5 nodule. The most experienced endocrinologists with over 10 years of post-residency experience, and comprising 66% of our sample, tended to avoid repeat FNA, preferring total thyroidectomy for EU-TIRADS 4 nodules instead. This is likely an example of “clinical inertia”, in which older Greek endocrinologists may not have adjusted their approach to thyroid nodule management, particularly regarding FNA before surgery [19]. For example, between 2007 and 2016, the indications for total thyroidectomy in Southwestern Greece were often unclear and suspicious FNA cytology for malignancy was documented in only 35.4% of cases prior to surgery. However, from 2011 to 2016, FNA use increased, indicating a shift toward more evidence-based decision-making and reduced instances of unjustified total thyroidectomy. In Greece, FNA is performed under ultrasound guidance by both endocrinologists and radiologists in both public and private sectors; however, we lack national data on the extent to which each group performs the procedure. Compared to physicians in the private sector, those in the public sector were more likely to choose repeat FNA for highly suspicious nodules, probably because aspirations are readily available at public hospitals and reimbursed by our healthcare system.

Molecular testing was chosen by only 20% of participants in the first clinical scenario and by 14% in the second. Jammah et al. reported that 47.3% of endocrinologists opted to repeat FNA in cases of AUS/FLUS (follicular lesion of indeterminate significance), while only 14.1% selected molecular testing due to its limited availability [20]. In contrast, a 2016 clinical survey in Italy found that molecular testing was the preferred approach for AUS/FLUS cytology. That study included 566 members of the Associazione Medici Endocrinologi (AME), with a response rate of 29.6% among respondents: 46% chose molecular testing followed by surgery if the result was positive, 43.6% preferred ultrasound follow-up due to the low malignancy risk, and only 9.3% recommended surgery, without providing further information [13]. Notably, the option to repeat FNA was not provided in that survey and only half of the participants had read the full guidelines. In addition, molecular testing is reported to be 2–5 times more likely in North America compared to other regions, possibly due to its being reimbursed by private insurance when clinically indicated [17]; nevertheless, in the aforementioned web-based survey, only 38.8% selected molecular testing for nodules with AUS/FLUS cytology. In Europe, commercial platforms for molecular testing of thyroid nodules are not routinely available. In Greece, only locally developed gene panels are accessible and only in the capital and the second-largest city. These panels are not covered by the national health system and information about their availability is limited. These factors significantly constrain Greek endocrinologists in utilizing molecular testing.

Regarding the surgical procedure for indeterminate solitary thyroid nodule, most Greek endocrinologists prefer total thyroidectomy over lobectomy. Fewer than 10% of endocrinologists chose lobectomy in either scenario, which is consistent with a previous report showing that this type of surgery is not widely practiced in Greece [19]. Notably, total thyroidectomy was preferred by 12% of clinicians for EU-TIRADS 4 nodules– particularly those with over 10 years of experience– and by 54% for EU-TIRADS 5 nodules. This preference for aggressive management of large or rapidly growing nodules mirrors a 20-year-old American survey conducted before FNA and molecular testing became widespread [21] supporting clinical inertia as a factor in current attitudes. High-resolution ultrasonography remains the best imaging modality for assessing malignancy risk, with the EU-TIRADS TR5 category showing the highest diagnostic accuracy according to a recent meta-analysis [22]. Studies by Trimboli et al. and Lu et al. reported malignancy rates of 87.7% and 80.4% for EU-TIRADS 5 nodules, far exceeding the 22% malignancy rate for Bethesda AUS/FLUS nodules [23, 24]. These findings may raise concerns about the safety and applicability of new approaches for patients with highly suspicious features for thyroid nodules measuring 2.5 cm. However, endocrinologists in the private sector and physicians in large cities are more likely to favor lobectomies. This may be because patients from rural areas are often referred to high-volume surgeons in cities due to a lack of local expertise, making a second completion surgery a logistical challenge.

Although the final question on reasons for non-adherence was not specific to the 2023 ETA guidelines for thyroid nodule management and was intended to apply broadly across all scenario clusters, it still offers valuable insight into general barriers to guideline adherence. Almost half of the responders expressed skepticism about the evidence, about one-third reported an inability to perform reliable ultrasound, approximately one-third cited the unavailability of molecular tests, and about one-fifth indicated a lack of proper information or a shortage of high-volume surgeons. In contrast, a survey of ATA members regarding the 2015 Adult Thyroid Nodule and Differentiated Thyroid Cancer Clinical Practice Guidelines reported that 83% of the responders strongly agreed that the guidelines were easy to apply in daily practice [14]. However, the survey did not assess how these guidelines influenced actual clinical decision-making. An important issue to consider is the discrepancy between physicians’ perceptions of guideline validity and their real-world decision-making. A more recent study by Schumm et al. demonstrated significant variation in guideline adoption and the extent of surgery or active surveillance [25]. Notably, physicians’ perception of 5- and 10-year cancer recurrence risk accounted for only 10.3% of the observed variance in decision-making for papillary thyroid cancer (PTC) patients.

This web-based survey has certain limitations. The final question addressing reasons for non-adherence to guidelines was, as mentioned, not specifically tailored to the 2023 ETA guidelines for the management of thyroid nodules. The limited availability of local laboratory panels for molecular genetic testing represents another constraint. The survey was conducted only 3 months after the publication of the ETA 2023 guidelines, which probably did not allow sufficient time for the guidelines to be widely incorporated into clinical practice. A further investigation should be conducted again after better assimilation of the 2023 ETA guidelines. However, the preference of experienced endocrinologists for total thyroidectomy over FNA repetition in the EU-TIRADS 4 and 5 categories with AUS cytology contradicts the recommendations of established scientific societies released over the past decade, such as the Bethesda 3rd edition and 2015 ATA guidelines. Additionally, the survey enrollment period lasted 5 months, potentially introducing bias. Participants responding closer to April 2024 may have had greater exposure to the guidelines compared to those who participated earlier. Changing long-established practices requires time and improved dissemination of guidelines and evidence-based data, facilitated through continuous medical education.

For clinical practice guidelines to achieve their purpose, their implementation is at least as important as their development and publication; however, this issue has received comparatively little attention. Numerous studies have highlighted the failure of clinicians to follow clinical practice guidelines [26, 27]. One of the main reasons for this is older age [26], as we also observed in the present survey. Clinicians in the latter half of their careers often practice based on personal experience rather than established guidelines. A thorough understanding of the reasons for physicians’ non-adherence to clinical guidelines is crucial for improving healthcare outcomes. Our findings underscore the urgent need to implement strategies that go beyond simply releasing guidelines. They are relevant not only for the latest ETA guidelines, but also for the upcoming ATA guidelines on thyroid nodules, specifically regarding their adoption in Europe or in other countries with healthcare landscapes similar to that of Greece. Systematically documenting the reasons for non-adherence is the obvious first and necessary step in understanding them in depth so that they can be properly addressed in order to optimize the management of thyroid nodules and improve patient outcomes.

## Conclusions

This web-survey revealed that many Greek endocrinologists deviate from the recently released 2023 ETA guidelines for management of thyroid nodules with AUS cytology. The current findings underscore the need for implementation of strategies alongside guideline dissemination.

## Electronic supplementary material

Below is the link to the electronic supplementary material.

Supplementary Material 1
