# Supplementary material for: Nationwide web survey on implementing the 2023 ETA guidelines for second-line management thyroid nodules with atypia of undetermined significance
Source: Hormones (Athens). 2025 Jul 28;24(4):1003–11. doi: 10.1007/s42000-025-00698-4 (PMC12678575; doi:10.1007/s42000-025-00698-4)
Supplement: Supplementary file 1 — Supplementary Material 1 [file 42000_2025_698_MOESM1_ESM.docx]

**Epidemiological data**

**1. Sex**

a. Male

b. Female

c. Other

**2. Age (years)**

a. 30-39

b. 40-49

c. 50-59

d. 60-69

e. >70

**3. Years since obtaining an endocrinology specialty**

a. 1-5

b. 6-10

c. 11-30

d. >30

**4. What is your workplace?**

a. Private sector

b. Public sector

**5. In which Prefecture do you practice your specialty? …………………………………………….**

**6. How confident do you feel about the management of patients with thyroid nodule or papillary thyroid carcinoma?**

a. Not at all confident

b. A little bit confident

c. Moderately confident

d. Very confident

e. Absolutely confident

**7. Which of the following do you think would help you manage patients with thyroid nodule or papillary thyroid carcinoma?**

a. Conferences

b. Clinical tutorials - case studies in tertiary hospitals

c. Bibliography

**Clinical scenarios**

**8. A 65-year-old woman has a non-functional solitary thyroid nodule, with dmax=2.5cm and EU-TIRADS 4 ultrasound standard (probability of malignancy 6-17%). Cytology revealed Atypia of Undetermined Significance (class III) by 2017 Bethesda (TBSRTC). What's your next step?**

a. repetition of FNA

b. molecular testing, if available

c. lobectomy

d. total thyroidectomy

**9. A 65-year-old woman has a non-functional solitary thyroid nodule, with dmax=2.5cm and EU-TIRADS 5 ultrasound standard (probability of malignancy 26-87%). Cytology revealed Atypia of Undetermined Significance (class III) by 2017 Bethesda (TBSRTC). What's your next step?**

a. repetition of FNA

b. molecular testing, if available

c. lobectomy

d. total thyroidectomy

**Exploring Reasons for non-adherence**

**What is/are the main reason(s) for non-adherence to the guidelines?**

A. Insufficient information

b. Skepticism about the guidelines and concerns for my patient's safety

c. Inability to perform a reliable neck ultrasound

D. Inability to conduct molecular testing

e. lack of experienced surgeons across Greece
